# A systematic investigation of the association between HPV and the clinicopathological parameters and prognosis of oral and oropharyngeal squamous cell carcinomas

**DOI:** 10.1002/cam4.1045

**Published:** 2017-04-04

**Authors:** Fengze Wang, Hui Zhang, Yang Xue, Jiao Wen, Jun Zhou, Xinjie Yang, Jianhua Wei

**Affiliations:** ^1^ State Key Laboratory of Military Stomatology & National Clinical Research Center for Oral Diseases & Shaanxi Clinical Research Center for Oral Diseases, Department of Oral and Maxillofacial Surgery, School of StomatologyThe Fourth Military Medical UniversityXi'anChina; ^2^ State Key Laboratory of Military Stomatology & National Clinical Research Center for Oral Diseases & Shaanxi Engineering Research Center for Dental Materials and Advanced Manufacture, Department of AnesthesiologySchool of StomatologyThe Fourth Military Medical UniversityXi'anChina; ^3^ State Key Laboratory of Military Stomatology & National Clinical Research Center for Oral Diseases & Shaanxi International Joint Research Center for Oral Diseases, Department of Oral Histology and PathologyThe Fourth Military Medical UniversityXi'anChina

**Keywords:** Human papillomavirus, immunohistochemistry, oral squamous cell carcinoma, oropharyngeal squamous cell carcinoma, P16, survival analysis

## Abstract

Human papillomavirus (HPV), the causal factor of cervical cancers, was closely linked to the etiology and prognosis of oropharyngeal squamous cell carcinoma (OPSCC), but its role in oral squamous cell carcinoma (OSCC) was unclear. In addition, few researches based on Chinese population were documented. Hence, we sought to investigate the relationship of HPV marker P16 protein to the clinicopathological parameters and survival of OPSCC and OSCC patients systematically to assess the influence of ethnic, regional difference on HPV susceptibility. Specimens from 93 OPSCC patients and 95 OSCC patients were recut, and P16 immunohistochemistry (IHC) was performed. Moreover, survival analysis was conducted to confirm the independent factors that influenced the prognosis. The P16 results were positive in 25.8% and 9.5% of patients with OPSCC and OSCC, respectively. The overall survival (OS) of HPV‐positive OPSCC patients was significantly longer than that of HPV‐negative OPSCC patients (*P* = 0.004). Conversely, statistical significance was not observed regarding the OS of OSCC patients (*P* = 0.343). Cox regression analysis indicated that T stage and P16 status were independent factors that affected the prognosis of OPSCC patients, and the smoking index influenced the prognosis of OSCC patients. Among OPSCC patients who received radiochemotherapy (RCT), HPV‐positive patients had a better survival rate than their HPV‐negative counterparts (*P* = 0.015). Conversely, no significant difference was observed between HPV‐positive and HPV‐negative OSCC patients who received RCT (*P* = 0.237). P16 is a credible surrogate by which to define HPV status. HPV expression had a favorable effect on OPSCC patients as opposed to their OSCC counterparts in this single center population‐based study.

## Introduction

Head and neck squamous cell carcinomas (HNSCCs) are ranked sixth among common cancers worldwide 1. Smoking and alcohol consumption are factors that increase the risk of development of head and neck cancers 2. However, in recent years, increased evidence has demonstrated that patients at high risk for human papillomavirus (HPV) exhibited an increase in the occurrence of head and neck cancers. In 1983, Stina Syrjanen revealed the existence of HPV 3. Additionally, HPV has been detected in the oropharynx, hypopharynx, and other areas 4. Human papillomavirus is a small, circular, double‐stranded DNA virus that is present in 26% of head and neck neoplasms 2. Moreover, HPV exhibits tissue tropism, cannot encode DNA polymerase and proliferates via host cell cycle proteins. The HPV genome exists in a free state, and its pathogenic genes, E6/E7, can be integrated into the patient's DNA 5. More specifically, the E6 protein can combine with cell cycle regulatory factor p53, resulting in its degradation and leading to malignant cell proliferation. The E7 protein binds to the retinoblastoma (Rb) gene, prompting the release of E2F, which gives rise to disorders of the cell cycle 6. Eventually, the HPV infection together with the prolonged accumulation of genetic damage results in malignant transformation of normal tissue.

Within head and neck cancers, the highest rate of HPV is found in oropharyngeal cancers, and this trend is increasing. HPV‐positive oropharyngeal squamous cell carcinoma (OPSCC) patients are distinctly different from their HPV‐negative counterparts in terms of pathogenesis, clinical manifestations and prognosis. For instance, the survival rate of HPV‐positive OPSCC patients is greater than that of patients with HPV‐negative cancers, particularly after RCT 7. Nevertheless, investigations regarding the relationship between HPV and oral squamous cell carcinoma (OSCC) are rare.

Currently, the methods used to detect HPV are different, consisting of nucleic acid in situ hybridization, gene chips and P16 immunohistochemistry (IHC), and the positive rates associated with the different methods are diverse 8. P16 IHC was first applied to detect HPV in cervical cancer as well as head and neck cancers 9, 10. Also, HPV was detected by HPV E6/E7 RNA detection and the results showed a high consistency with P16 IHC 11. Moreover, in paraffin‐embedded specimens rather than fresh tissues, HPV detection sensitivity may reduce owing to DNA and RNA degradation 12. Consequently, we used P16 IHC to detect HPV and consulted the medical records of 188 OSCC and OPSCC patients to analyze the relationship between P16 status and clinicopathological parameters. We evaluated the effect of P16 status on overall survival (OS) using a survival analysis to provide useful prognostic indicators and theoretical support.

## Patients and Methods

A total of 188 OPSCC patients were included in this retrospective study from January 2007 to December 2013. The criteria for patient inclusion were as follows: (1) patients diagnosed with squamous cell carcinoma based on hematoxylin‐eosin staining (H&E); (2) availability of complete paraffin sections; and (3) detailed information of medical records and follow‐up data. All pathological specimens were collected at the Department of Oral and Maxillofacial Surgery, School of Stomatology, the Fourth Military Medical University (FMMU). All cancers were assessed via the 2002 American Joint Committee on Cancer staging system. All patients were followed up by phone, mail, and medical records. This study was approved by the Medical Ethics Committee of FMMU.

### P16 immunohistochemical staining

The samples were recut to conduct P16 IHC. After dewaxing hydration, we used a 0.01 mol/L sodium citrate buffer with a heat‐based method to retrieve the antigen. Then, we exposed the samples to the mouse anti‐human monoclonal antibody of P16 (1:50; Abcam, Cambridge, USA) and incubated them at 4°C overnight. The next day, a secondary antibody kit (SPlink Detection Kits, SP9002, ZSGB‐BIO, Beijing, China) was employed, and a freshly prepared DAB dilution was used to display the color.

All the immunohistochemical results were determined by two experienced pathologists using two methods. The criteria of positive staining were a blue nucleus and brown cytoplasm staining. For detailed methods, please see the study by Shamir P. Chandarana 13.

### Statistical analysis

SPSS19.0 (IBM, Armonk, NY) software was used for the statistical analysis. Fisher's exact test or the chi‐square test were used to analyze data from independent groups. The OS was calculated in months and ranged from diagnosis to death due to any reason or censoring on September 1, 2016. Kaplan–Meier curves and log‐rank methods were used to perform survival analyses, and significant differences between the two curves were determined. Subsequently, we conducted a multivariate Cox regression analysis to determine the independent prognostic factors. A *P* < 0.05 was considered statistically significant.

## Results

### The expression of P16 in OPSCC and OSCC samples

**Figure 1:**
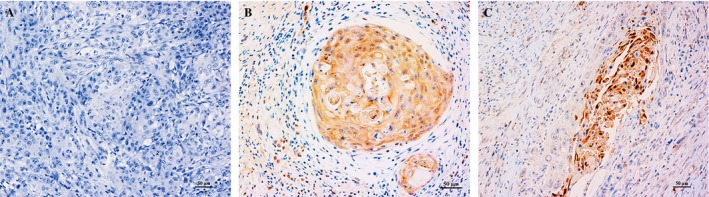
P16 IHC of oropharyngeal squamous cell carcinoma (OPSCC) and oral squamous cell carcinoma (OSCC) samples. (A) Negative expression of P16 (IHC ×200); (B) Positive expression of P16 in an OPSCC sample (IHC ×200); (C) Positive expression of P16 in an OSCC sample (IHC ×200).

The immunohistochemical results are shown in Figure 1. The expression of P16 was positive in the cytoplasm in OPSCC and OSCC samples. The positive ratios were 25.8% (24/93) in OPSCC and 9.5% (9/95) in OSCC samples.

### Comparison of clinical parameters according to P16 status

For OPSCC patients, age, smoking index, histotype, N stage, and TNM clinical stage were statistically significant when the P16‐positive group was compared to their P16‐negative counterparts (*P* < 0.05, See Table 1). However, a higher proportion of patients were female and T1 + T2 stage patients, although the difference was not statistically significant (*P* > 0.05). Additionally, for OSCC patients, the smoking index, N stage and TNM clinical stage were statistically significant compared to the P16‐negative group (*P *<* *0.05, See Table 1). However, a higher proportion of patients were male, older than 60 years of age, had a smoking index <20 and were T3 + T4 stage patients, although significance was not achieved.

**Table 1:** Relation between clinicopathological parameters and P16 status for oropharyngeal squamous cell carcinoma (OPSCC) and oral squamous cell carcinoma (OSCC)

Variable	Number	P16 status of OPSCC		*P*	Number	P16 status of OSCC		*P*
		−	+			−	+	
Gender								
Male	75	56 (81.2)	19 (79.2)	0.831	55	49 (57.0)	6 (66.7)	0.729
Female	18	13 (18.8)	5 (20.8)		40	37 (43.0)	3 (33.3)	
Age								
<60	48	31 (44.9)	17 (70.8)	0.029*	56	51 (59.3)	5 (55.6)	0.828
≥60	45	38 (55.1)	7 (29.2)		39	35 (40.7)	4 (44.4)	
Smoking⊗								
<20	54	35 (50.7)	19 (79.2)	0.015*	37	30 (58.1)	7 (77.8)	0.026*
≥20	39	34 (49.3)	5 (20.8)		58	56 (41.9)	2 (22.2)	
Site								
TR✩	49	36 (52.2)	13 (54.2)	0.341		/	/	
PT∆	9	5 (7.2)	4 (16.7)			/	/	
SP∇	35	28 (40.6)	7 (29.2)			/	/	
HP□		/	/		19	19 (22.1)	0 (0.0)	0.136
Cheek		/	/		22	21 (24.4)	1 (11.1)	
Tongue		/	/		28	25 (29.1)	3 (33.3)	
MF○		/	/		26	21 (24.4)	5 (55.6)	
Histotype◊								
P/M	33	20 (29.0)	13 (54.2)	0.026*	16	13 (15.1)	3 (33.3)	0.174
W	60	49 (71.0)	11 (45.8)		79	73 (84.9)	6 (66.7)	
T stage								
1–2	69	50 (72.5)	19 (79.2)	0.518	53	46 (53.5)	7 (77.8)	0.290
3–4	24	19 (27.5)	5 (20.8)		42	40 (46.5)	2 (22.2)	
N stage								
N0	41	35 (50.7)	6 (25.0)	0.029*	16	12 (14.0)	4 (44.4)	0.041*
N+	52	34 (49.3)	18 (75.0)		79	74 (86.0)	5 (55.6)	
TNM								
I–II	44	37 (53.6)	7 (29.2)	0.039*	30	24 (27.9)	6 (66.7)	0.025*
III–IV	49	32 (46.4)	17 (70.8)		65	62 (72.1)	3 (33.3)	

⊗: Smoking index: pack‐year; ✩: TR: Tongue root; ∆:PT: Palatine tonsil; ∇: SP: Soft palate; □: HP: Hard palate; ○: MF: Mouth floor; ◊: P: Poor differentiation; M: moderate differentiation; W: well differentiation; *: Chi‐square test, *P* < 0.05.

### Survival analysis according to P16 status

**Figure 2:**
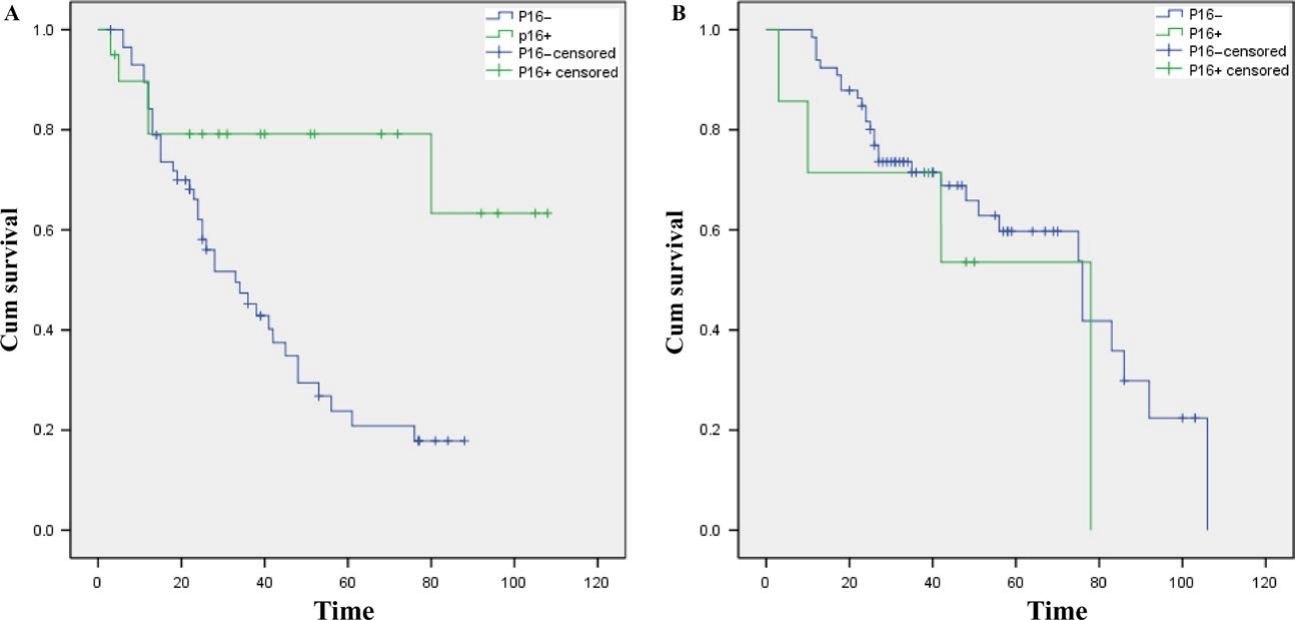
Overall survival (OS) of patients with oropharyngeal squamous cell carcinoma and oral squamous cell carcinoma according to P16 status. (A) Those with P16 expression have significant improved OS (*P* = 0.004). (B) Those with P16 negative expression have slight improved OS (*P* = 0.343).

The OS was calculated in months and ranged from diagnosis to death due to any reason or censoring on September 1, 2016. The survival time ranged from 3 months to 108 months for OPSCC patients and from 3 months to 106 months for OSCC patients. Moreover, median follow‐up time was 28 months (3–108 months) and 36 months (3–106 months) in OPSCC and OSCC, respectively. At 5 years, P16‐positive OPSCC patients had an OS of 79% versus 21% in P16‐negative patients (See Fig. 2). OPSCC patients with a P16‐positive status had longer OS than the P16 negative group (log‐rank *P*‐value: 0.004, Fig. 2). However, no statistical significance was observed between the two curves within the OSCC group (log‐rank *P*‐value: 0.343, Fig. 2). At 5 years, the P16‐negative OSCC group had an OS of 59.7% versus 53.6% in the P16‐positive group.

The univariate analysis revealed that histotype, P16 status, and T stage were relevant factors that influenced OPSCC prognosis (*P* < 0.05). Multivariate Cox regression analysis showed that T stage and P16 were independent prognostic factors (*P* < 0.05, See Table 2). Moreover, in OSCC patients, the univariate analysis showed that T stage, TNM stage, age, and smoking index were statistically significant (*P *<* *0.05). The results of the Cox regression model showed that the smoking index was an independent factor that affected the prognosis of OSCC patients (See Table 2).

**Table 2:** Multivariable analysis of OS (Cox regression model)

Variables	Overall survival (OPSCC)		Overall survival (oral squamous cell carcinoma (OSCC))	
	HR (95% CI)	*P*	HR (95% CI)	*P*
Age	/	/	1.835 (0.874–3.856)	0.109
TNM stage	/	/	0.383 (0.073–2.014)	0.257
Smoking	/	/	0.386 (0.178–0.838)	0.016^a^
T stage	0.489 (0.261–0.917)	0.026^a^	1.012 (0.202–5.071)	0.988
Histotype	1.105 (0.603‐2.026)	0.747	/	/
P16 status	3.856 (1.502–9.899)	0.005^a^	/	/

a Chi‐square test, *P*<0.05.

### Sensitivity of HPV‐driven OPSCC and OSCC to RCT

**Figure 3:**
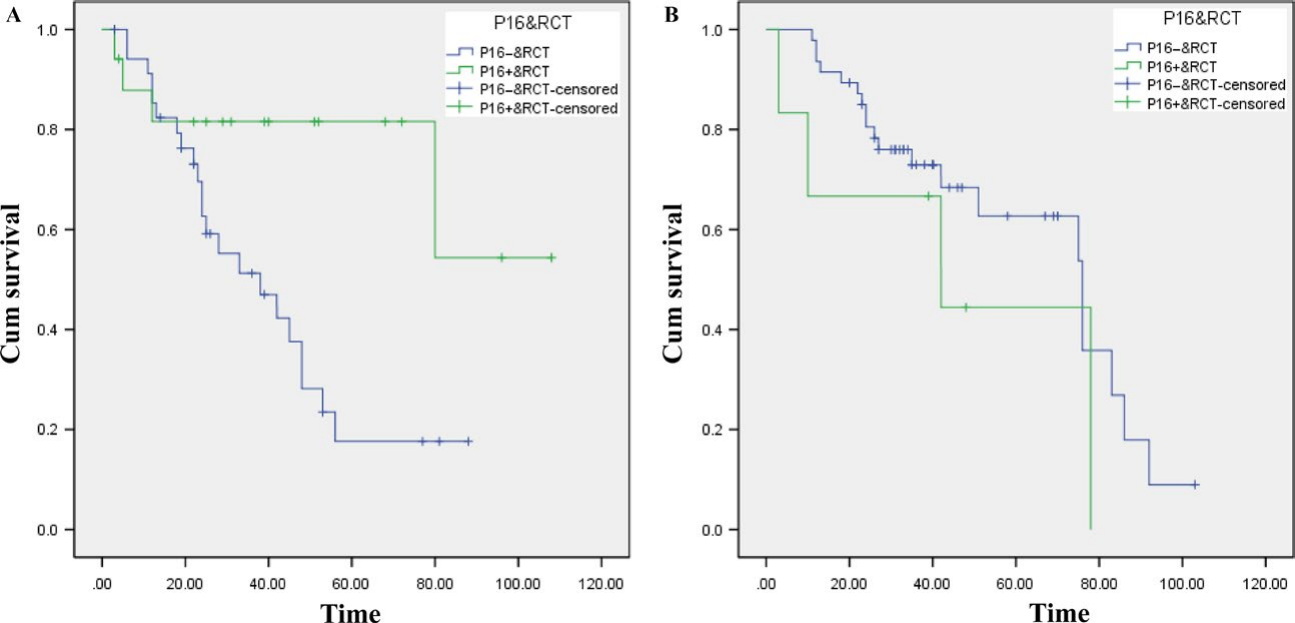
Sensitivity of P16‐positive and ‐negative oropharyngeal squamous cell carcinoma (OPSCC) and oral squamous cell carcinoma (OSCC) to RCT. (A) Those with P16 expression OPSCC have an improved sensitivity to postoperative RCT (*P* = 0.015); (B) Those with P16‐negative OSCC have an improved sensitivity to postoperative RCT (*P* = 0.237).

A growing body of evidence supports the idea that HPV‐driven OPSCC is more radiosensitive than HPV‐negative OPSCC. However, studies on Chinese individuals are relatively rare. Hence, we investigated the sensitivity of HPV‐driven OPSCC and OSCC to RCT. We concluded that HPV‐driven OPSCC patients were more sensitive to RCT than their counterparts, and the difference was statistically significant (*P* = 0.015, See Fig. 3A). At 5 years, P16‐positive OPSCC patients who received RCT after surgery had an OS of 80% versus their counterparts, who had an OS of 19% (See Fig. 3A). However, with regard to OSCC, the two overall survival curves were not significantly different (*P* = 0.237, See Fig. 3B). Similarly, at 5 years, P16‐positive patients who were exposed to RCT had an OS of 42% versus their counterparts, who had an OS of 63% (See Fig. 3B).

## Discussion

Human papillomavirus is a causal factor of cervical cancer and several types of HNSCC, especially OPSCC 2. We chose cyclin‐dependent kinase inhibitor (CDKI) P16 protein, which is encoded by CDKN2A, as a surrogate to detect HPV expression in OPSCC and OSCC specimens. The positive rate of P16 in OPSCC was 25.8%, which was inconsistent with previous reports 14, 15. The possible reasons were that P16 positivity may influenced by regional, individual, and sexual culture difference.

For OPSCC patients, clinicopathological parameter analysis showed that the expression of P16 was associated with age, smoking index, histotype, N stage, and TNM stage (*P* < 0.05). In addition, P16‐positive OPSCC patients were more often younger than 60 years of age than P16‐negative patients (70.8% vs. 44.9%, respectively). Other differences in these patients included the following: smoking index <20 (79.2% vs. 50.7%, respectively), poor/moderate differentiation (54.2% vs. 29.0%, respectively), lymph node metastasis (75.0% vs. 49.3%, respectively), and clinical stage of III–IV (70.8% vs. 46.4%, respectively). These results are consistent with domestic and international scientific reports 16. Although the T stage was not significantly different (*P* < 0.05), which is inconsistent with current reports 16, there was a tendency that a higher proportion of patients were in the T1 + T2 stages (79.2% vs. 72.5%, respectively). Likewise, for OSCC patients, P16 status was closely associated with a smoking index <20 (77.8% vs. 58.1%), N0 stage (44.4% vs. 14.0%), and clinical stage of I–II (66.7% vs. 27.9%) (*P* < 0.05). It is noteworthy that our research showed that male patients were vulnerable to HPV infection, which is consistent with some studies 12, 17, but inconsistent with other studies 2, 18, 19. Additionally, patients older than 60 years of age were susceptible to HPV, which was not supported by other reports 20. Moreover, the P16‐positive group had more patients whose smoking index was <20, which was inconsistent with one study 17. The possible cause of this result was the diversity of the stratified criteria for the smoking index. In terms of tumor differentiation, P16‐positive patients usually showed poor and moderate differentiation, which was consistent with a current report 12, but showed discrepancies with other research 18, 19. The possible reason for this inconsistency was that the ratio of well‐differentiated OSCC in the studies mentioned above was relatively larger than our data, contributing to selection bias. In addition, our results revealed that N0 stage and clinical stage I–II were closely correlated with P16 status (*P* < 0.05), and a higher proportion of OSCC patients were staged T1 and T2 in the P16‐positive group than in the P16‐negative group, which was consistent with current evidence 19, but inconsistent with results from other studies 21, 22. The possible reason for this inconsistency was that the P16 protein plays a role in inhibiting the carcinogenesis, metastasis, and development of OSCC, which could be deemed as a significant marker of early detection and prognostic evaluation. To sum up, HPV is an established factor that influences the development of OPSCC, and the relevant research was sufficient to determine the underlying mechanism. In contrast, research regarding the relationship between HPV and OSCC is rare. Hence, numerous population‐based investigations are needed to provide evidence for treatment methods for OSCC and OPSCC.

Furthermore, we evaluated the effect of clinicopathological parameters and P16 status on OS via a survival analysis and used the log‐rank test to compare the two Kaplan–Meier curves. The results showed that for OPSCC patients, the OS of the P16‐positive group was significantly higher than that of their counterparts (*P* = 0.015), which was consistent with other studies 23, 24. Multivariate Cox regression analysis indicated that P16 status and T stage were independent prognostic factors, which was consistent with international research 7, 25. Furthermore, for OSCC patients, the OS of the P16‐negative group was slightly higher than that of the P16‐positive group, but not statistically significant (*P* = 0.343); these findings were consistent with findings from another study 26. However, controversial results could be found with respect to the relationship between P16 status and the prognosis of OSCC 27, 28, 29, possibly due to individual differences, the strategy, and data that were lost to follow‐up, suggesting that more systematic research is needed to verify these results. The Cox regression model revealed that the smoking index is an independent prognostic factor. However, Loeschke et al. 28 used Cox regression analysis to demonstrate that N stage was a prognostic factor.

In addition, we determined that for OPSCC patients, the P16‐positive and RCT groups exhibited improved OS compared to their counterparts. The log‐rank test showed that the two Kaplan–Meier curves were significantly different (*P* = 0.015), which was consistent with current studies 7, 30. However, for OSCC patients, the P16‐negative and RCT groups showed improved OS compared to their counterparts, but the Kaplan–Meier curves were not significantly different (*P* = 0.237).

The study had evident advantages and limitations. The primary strength was systematic large sample research including medical records collection, HPV detection, and survival analysis based on Chinese population. It is worth noting that in the year of 2016, the first HPV vaccine (Cervarix) has been licensed to prevent cervical cancers in China. Increasing importance has been attached to HPV carcinogenesis in China. Moreover, girls and women aged between 9 and 25 were recommended to vaccinate. Most strikingly, our research showed that female patients with OPSCC were vulnerable to HPV infection. Taking account of the rare reports based on Chinese population, our single center's systematic investigation will offer support and guidance to future utilization of HPV vaccine to prevent OPSCC. One limitation is that p16 IHC could be influenced by many elements like PH and temperature. In addition, DNA and RNA may degrade in paraffin‐embedded specimen. Hence, we will conduct a prospective study to collect fresh tissues to complete HPV E6/E7 mRNA detection and P16 IHC to increase the credibility of our research. The Secondly, there were a relative lower P16 prevalence in OSCC, which caused insufficient power and deviation of statistical analysis. We believed that this bias could be reduced by increasing sample capacity and multicenters’ cooperation.

In conclusion, P16 status could be used as a crucial marker to provide evidence for prognosis and future treatment, resulting in its emergence as a hotspot of research worldwide. P16 positivity was influenced by regional and ethnic difference. We believe that the study at present might be beneficial to the future research with increased sample size and larger number of participants.

## Conflict of Interest

None declared.
